# Inhibition of endoplasmic reticulum stress is involved in the neuroprotective effect of aFGF in neonatal hypoxic-ischaemic brain injury

**DOI:** 10.18632/oncotarget.17524

**Published:** 2017-04-29

**Authors:** Yingying Hu, Zhouguang Wang, Shulin Pan, Mingchu Fang, Huai Jiang, Yuqin Mao, Hao Zhang, Yiming Ji, Fabiao Zhang, Li Lin, Zhenlang Lin, Jian Xiao

**Affiliations:** ^1^ Department of Neonatology, The Second Affiliated Hospital and Yuying Children's Hospital, Wenzhou Medical University, Wenzhou, Zhejiang, 325027, China; ^2^ Molecular Pharmacology Research Center, School of Pharmaceutical Science, Wenzhou Medical University, Wenzhou, Zhejiang, 325035, China; ^3^ Department of Hepatobiliary Surgery, Taizhou Hospital of Zhejiang Province, Wenzhou Medical University, Linhai, 317000, China

**Keywords:** acidic fibroblast growth factor, neonatal hypoxic-ischaemic brain injury, endoplasmic reticulum stress, intranasal

## Abstract

Acidic fibroblast growth factor (aFGF) has been shown to exert neuroprotective effects in experimental models and human patients. In this study, we investigated whether aFGF intranasal-treatment protected against neonatal hypoxic-ischaemic brain injury and evaluated the role of endoplasmic reticulum stress. The Rice-Vannucci model of neonatal hypoxic-ischaemic brain injury was used in 7-day-old rats, which were subjected to unilateral carotid artery ligation followed by 2.5 h of hypoxia. Intranasal aFGF or vehicle was administered immediately after hypoxic-ischaemic injury (100 ng/g) and then twice a day for 1 week to evaluate the long-term effects. Here we reported that intranasal-treatment with aFGF significantly reduced hypoxic-ischaemic brain infarct volumes and the protective effects were at least partially via inhibiting endoplasmic reticulum stress. In addition, aFGF exerted long-term neuroprotective effects against brain atrophy and neuron loss at 7-day after injury. Our data indicate that therapeutic strategies targeting endoplasmic reticulum stress may be promising to the treatment of neonatal hypoxic-ischaemic brain injury.

## INTRODUCTION

Neonatal encephalopathy due to perinatal hypoxia-ischemia (HI) is a devastating disease that is a main cause of morbidity and mortality in infants and children, with a reported incidence of 2–9 cases per 1000 births [1]. Furthermore, 20–50% of infants who suffer from perinatal HI injury die during the newborn period, and up to 25% lead to long-term neurological deficits [2]. Currently, effective treatments for attenuating HI injury in the clinical setting are limited. Thus, understanding the molecular mechanisms may provide new insights into its pathogenesis and treatment for neonatal HI brain injury.

The endoplasmic reticulum (ER) is a cell compartment that is responsible for proper folding of secretory and transmembrane proteins in an energy-dependent manner. Conditions that interfere with ER function cause accumulation of unfolded proteins in the ER lumen, which is known as ER stress, and activate a homeostatic signalling network known as the unfolded protein response (UPR) [3–5]. The UPR aims to restore ER function, but when stress is extensive or sustained, ER function cannot be restored and eventually leads to the removal of affected cells by apoptosis [6, 7]. In unstressed cells, the ER chaperone glucose-regulated-protein-78 (GRP-78) binds pancreatic-ER-kinase (PERK), activating-transcription factor-6 (ATF-6) and inositol-requiring enzyme-1 (IRE-1), which keeps them inactive [8]. Upon onset of stress, ER calcium depletion and, thus, accumulation of unfolded proteins, GRP-78 is drawn away from PERK, ATF-6 and IRE-1 by binding these unfolded proteins, leading to activation of UPR actors, such as ATF-6, CHOP, PDI, XBP-1, etc [9]. ER stress plays a critical role in mediating a range of diseases, including ischaemia/reperfusion injury [10, 11], neurodegeneration [12, 13] and diabetes [14, 15], which indicates that ER stress is a probable instigator of pathological cell death and dysfunction.

Acidic fibroblast growth factor (aFGF or FGF-1) is a multipotent factor associated with cell proliferation, differentiation and survival during development via initiating various intracellular signal transduction pathways [16, 17]. Studies have identified neuroprotective roles of aFGF in animal models of cerebral ischaemia /reperfusion injury. As Hossain reported, aFGF substantially protects the neonatal brain against neuronal injury induced by the NMDA receptor agonist quinolinic acid [18], which is considered a model of the relatively late events associated with HI brain injury [19, 20]. A previous study found that the neuroprotective effects exerted by aFGF against excitotoxicity are mediated by activation of the PI3K/Akt-dependent and MAPK/CREB-independent signalling pathways [21]. Moreover, transgenic expression of human FGF-1 protects against HI brain injury via intervening in caspase-XIAP signalling cascades in a rat model of perinatal HI injury [22].

In this study, we investigated the effects of aFGF and the mechanisms underlying the development of ER stress in neonatal HI brain injury. We found that intranasal-treatment with aFGF exerted neuroprotective effects against neonatal HI brain injury and that the effect was related to the inhibition of ER stress both *in vivo* and *in vitro*. Collectively, aFGF suggests to being an effective and feasible target for the development of drugs intended to treat neonatal HI brain injury*.*

## RESULTS

### aFGF protects the brain from infarction and improves morphological recovery after HI injury

**Figure 1:**
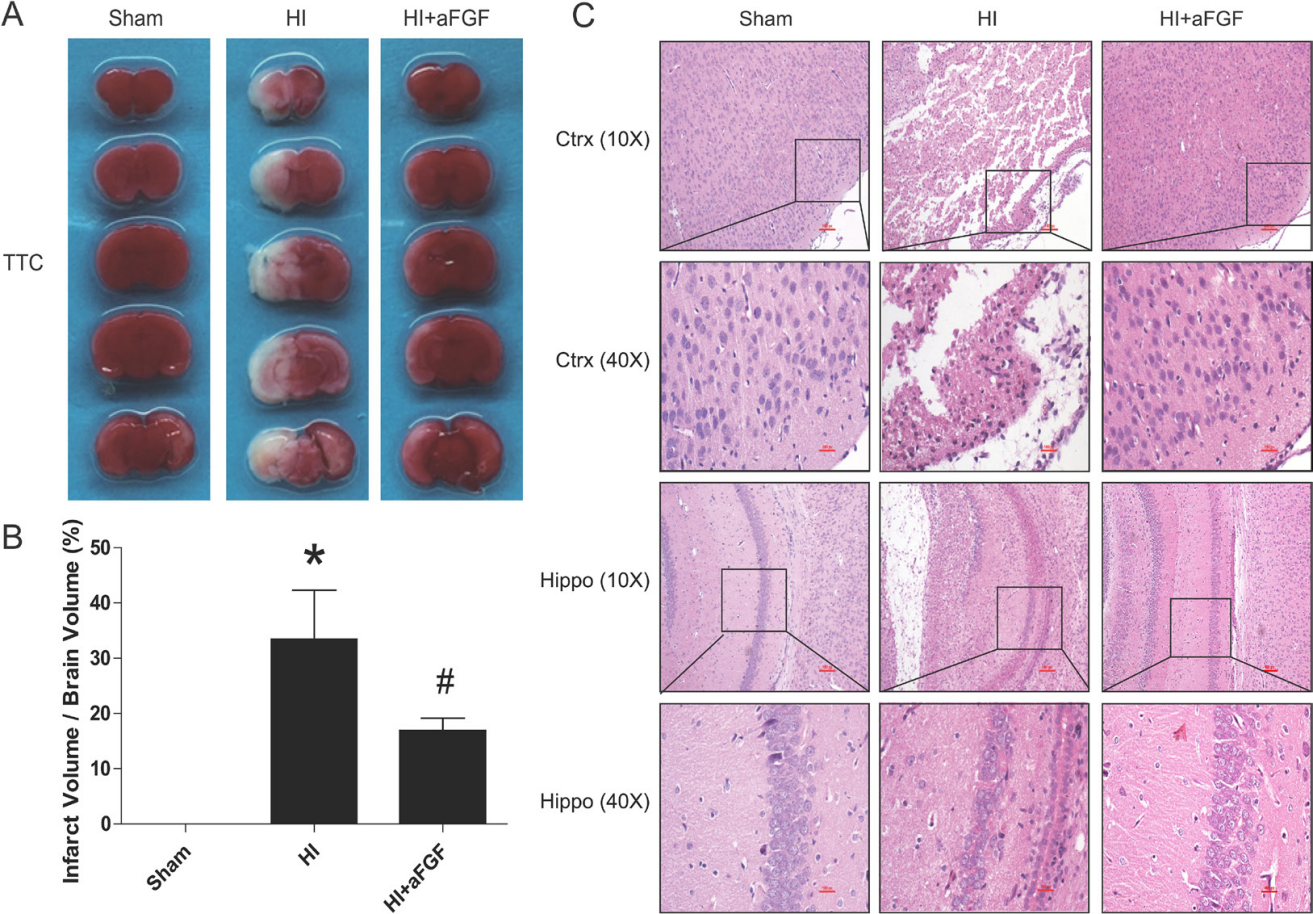
aFGF reduces infarct volumes and improves morphological recovery (**A**) Representative TTC-stained coronal brain sections from the sham, HI and aFGF-treated groups at 24 h after HI. (**B**) Quantitative analysis of infarct rate from A. *p < 0.05 versus the sham group, ^#^*p* < 0.05 versus the HI group. (**C**) Representative HE staining pictures of the frontal cortex and dorsal hippocampus from the lesioned (ipsilateral) side at 1 week after HI are shown. Scale bar = 400 μm and 100 μm (selected boxes). Data are expressed as the mean value ± SEM, *n* = 5 rats per group.

At 24 h after neonatal HI brain injury, we determined the infarct volume via TTC staining. aFGF significantly reduced the mean infarct volume in the aFGF-treated group (17 ± 2%) compared with the HI group (33 ± 9%) (mean ± SEM, Figure 1A, 1B). We further compared histopathological alterations in the cortex and hippocampus at 7 d after HI brain injury via HE staining. Brains from the sham group appeared normal structure, regular cell arrangements and complete cell outlines with round, intact nuclei. In contrast, abnormal cell arrangements, shrunken cells with pyknotic nuclei, as well as decreased cell density and even partial or complete degeneration of neurons, were observed in the cortex and hippocampus regions of the HI group at 7 d after injury. Partial recovery was observed in the aFGF-treated group (Figure 1C).

### aFGF attenuates HI injury-induced neuronal cell apoptosis

**Figure 2:**
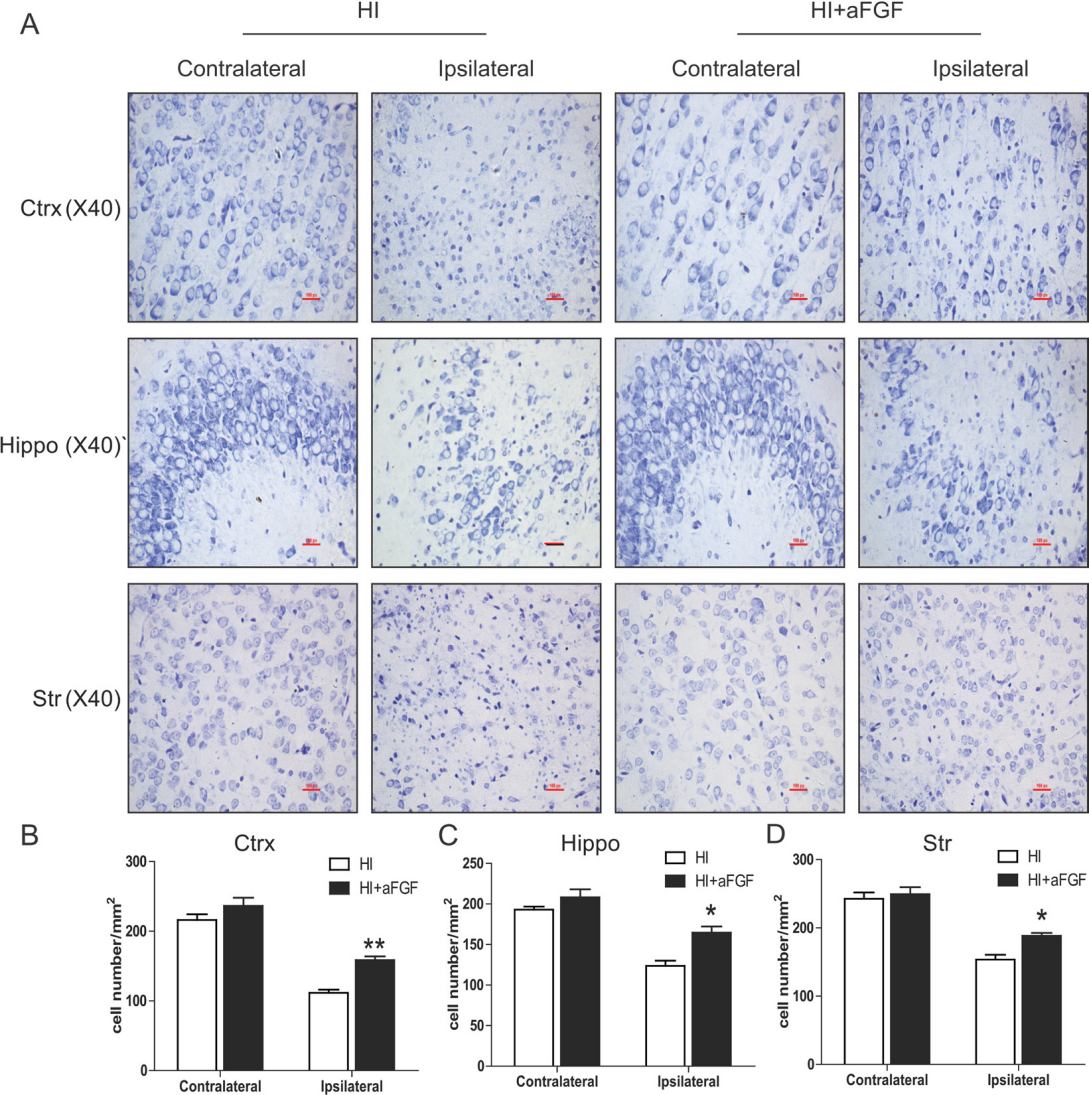
aFGF attenuates HI-induced neuronal cell death Coronal brain sections were obtained from the sham, HI and aFGF-treated-group at 1 week after HI injury and stained with Nissl solution. (**A**) Representative pictures of the frontal cortex, dorsal hippocampus and striatum from the lesioned (ipsilateral) and unlesioned (contralateral) sides are shown. Scale bar = 100 μm. (**B**), (**C**) and (**D**) Analysis of Nissl data from A. *p < 0.05, **p < 0.01 versus the HI group. Data were expressed as the mean value ± SEM, *n* = 5 rats per group.

As the Nissl staining shown, a marked increase in the number of degenerated and even dying neurons characterized by shrinkage of the cytoplasm, pyknosis of the nuclei and absence of Nissl bodies were seen throughout the ipsilateral cerebral cortex, hippocampus and striatum, but not in the contralateral brain, after brain injury induced by left carotid ligation and hypoxia. However, neuronal cells were substantially protected by aFGF administration immediately after HI injury (Figure 2).

**Figure 3:**
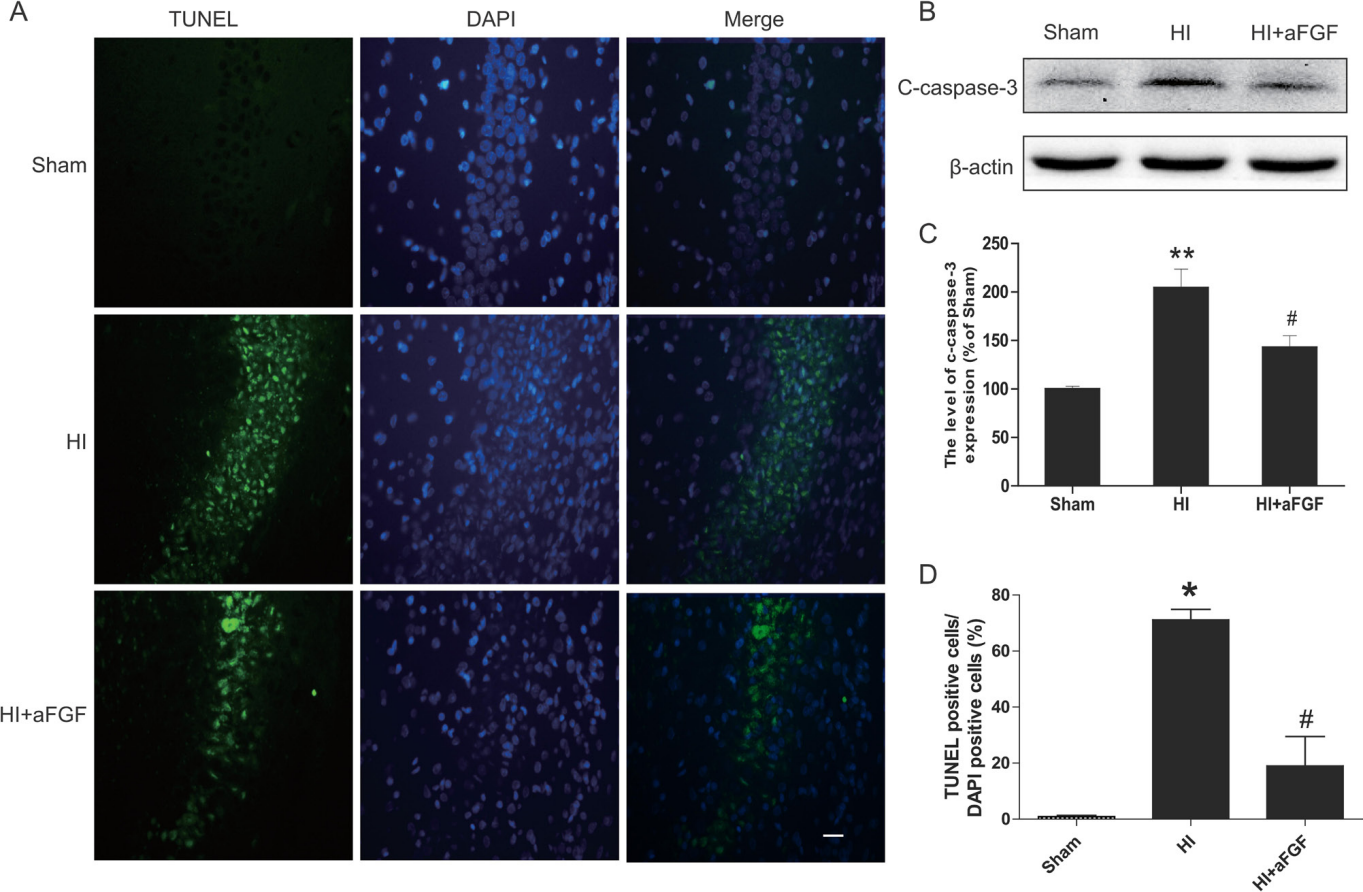
aFGF decreases the level of apoptosis in HI brain injury (**A**) Representative TUNEL-stained (*green*) and DAPI-stained (*blue*) brain sections in the hippocampus at 1 week post-injury. Scale bar = 100 μm. (**B**) Detection of apoptosis proteins was performed via western blotting. The protein expression levels of cleaved caspase 3 in the brains of sham rats, HI rats and HI rats treated with aFGF. (**C**) Quantification of western blot data from B. **p < 0.01 versus the sham group, ^#^*p* < 0.05 versus the HI group. (**D**) Quantitative analysis of TUNEL staining data from A. *p < 0.05 versus the sham group, ^#^*p* < 0.05 versus the HI group. Data are expressed as the mean value ± SEM, *n* = 5 rats per group.

TUNEL staining provided us with knowledge about the cell apoptosis based on DNA breaks. In the hippocampus, few TUNEL-positive cells were observed in the sham animals at 7 d after HI injury, whereas the number of TUNEL-positive cells increased significantly in the HI group compared to the sham group. In contrast, significantly reductions in the numbers of apoptotic cells were noted in the aFGF-treated group in comparing to the HI group (Figure 3A, 3D). Moreover, cleaved caspase 3, which is the terminal executing enzyme for cleavage of substrate, was measured by western blot analysis 24 h after HI to examine the effects of HI and aFGF on neuronal apoptosis. The expression of cleaved caspase 3 increased significantly after HI injury which suggested the enhanced apoptosis. aFGF intervention obviously inhibited the HI-induced up-regulation of cleaved caspase 3 in the brain (Figure 3B, 3C). These results indicated that the neural protective effects of aFGF might be at least partially through attenuating apoptosis.

### aFGF inhibits activation of ER stress after HI injury

**Figure 4:**
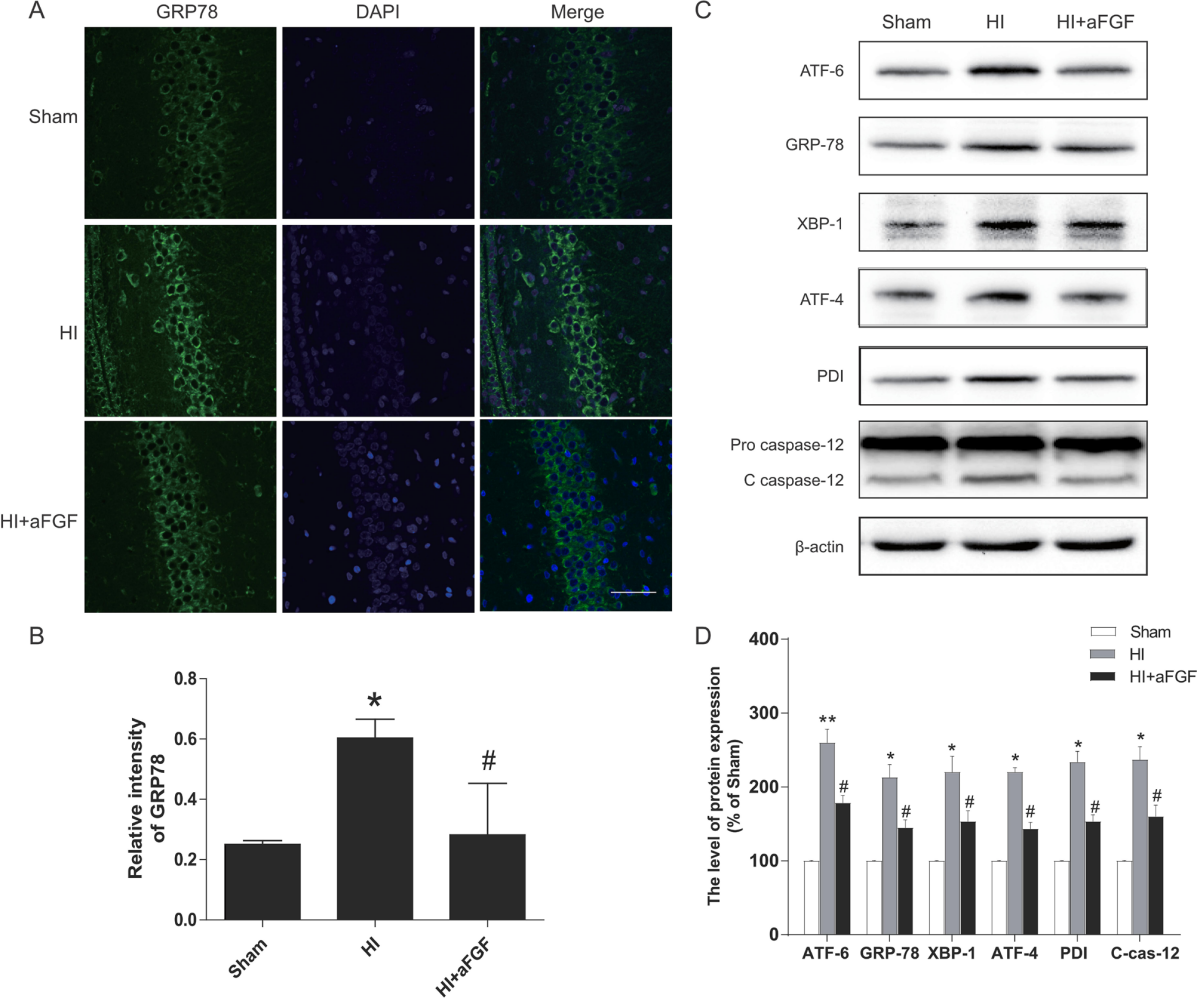
HI leads to activation of ER stress markers at 24 h after injury, and aFGF significantly attenuates these effects Rats subjected to HI injury were immediately treated with aFGF (100 ng/g). Coronal brain sections were prepared at 24 h post-injury. (**A**) Representative immunofluorescence staining results for GRP-78 (*green*) in the hippocampus; nuclei were labelled with DAPI (*blue*). Scale bar = 75 μm. (**B**) Quantitative analysis of the immunofluorescence data from A. *p < 0.05 versus the sham group, ^#^*p* < 0.05 versus the HI group. (**C**) Representative western blots of the ER stress markers ATF-6, GRP-78, XBP-1, ATF-4, PDI and cleaved caspase 12 in the sham, HI model and HI model + aFGF treatment groups. (**D**) Quantification of western blot data from C. *p < 0.05, **p < 0.01 versus the sham group, ^#^*p* < 0.05 versus the HI group. Data are expressed as the mean value ± SEM, *n* = 5 rats per group.

**Figure 5:**
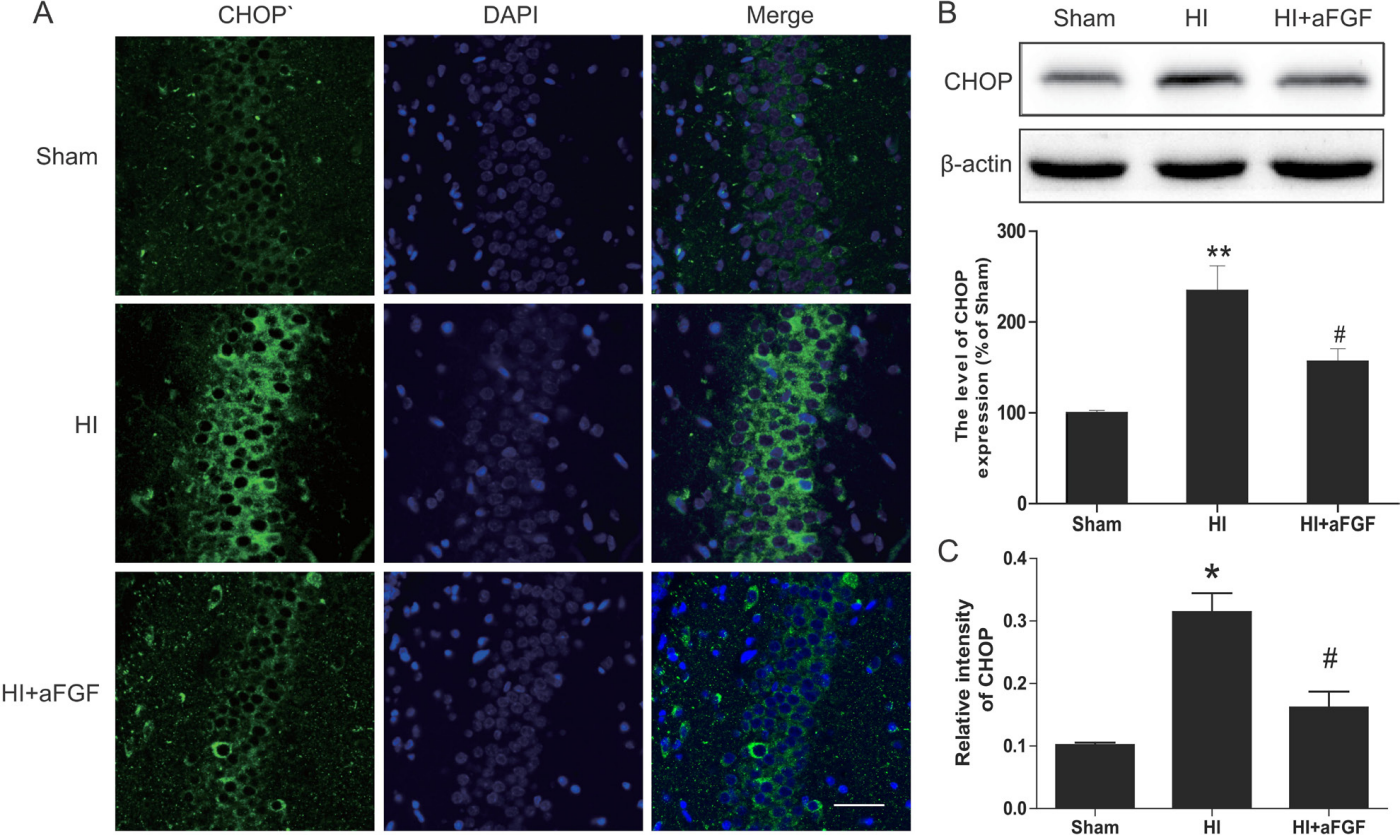
aFGF inhibits ER stress-related CHOP expression after HI (**A**) Representative immunofluorescence staining results for CHOP (*green*) in the hippocampus; nuclei were labelled with DAPI (*blue*). Scale bar = 75 μm. (**B**) Representative western blots and quantification data for CHOP in the sham, HI model and HI model + aFGF treatment groups. **p < 0.01 versus the sham group, ^#^*p* < 0.05 versus the HI group. (**C**) Quantitative analysis of immunofluorescence data from A. *p < 0.05 versus the sham group, ^#^*p* < 0.05 versus the HI group. Data are expressed as the mean value ± SEM, *n* = 5 rats per group.

A previous work demonstrated that the UPR was strongly activated after neonatal HI brain injury [23]. To test the extent of ER dysfunction after HI brain injury and the effects of aFGF on ER stress, several ER stress markers were measured 24 h after HI injury. As the western blotting results shown, the levels of ATF-6, GRP-78, XBP-1, ATF-4, PDI, cleaved caspase 12 and CHOP were high in the HI group, whereas post-treatment with aFGF decreased significantly these ER stress markers expression after HI compared with the HI group (Figure 4C, 4D and Figure 5B). Besides, immunofluorescent staining in the hippocampus revealed more GRP-78 (Figure 4A, 4B) and CHOP (Figure 5A, 5C) signals in the HI group when compared to the sham. However, GRP78-positive and CHOP-positive cells were reduced in the aFGF group versus the HI group. These results indicated that aFGF effectively inhibited ER stress-related protein expressions in the brain after HI injury.

### Long-term effects of aFGF treatment

**Figure 6:**
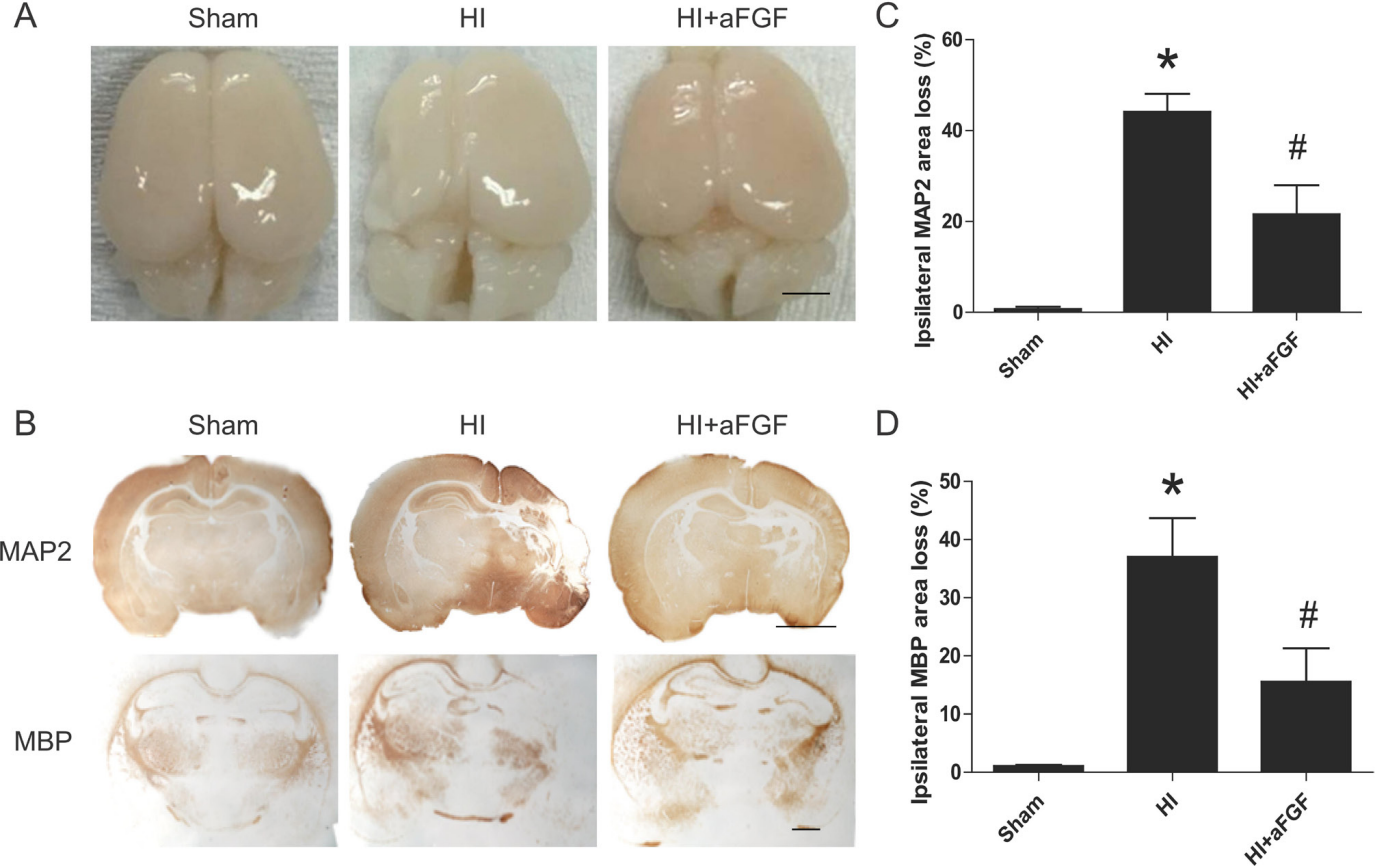
Long-term effects of aFGF (**A**) Top view of the brains from the sham, HI and aFGF-treated-groups at 1 week after HI injury. (**B**) Representative sections of MAP-2 and MBP area loss. (**C**) Quantification of ipsilateral MAP-2 area loss. *p < 0.05 versus the sham group, ^#^*p* < 0.05 versus the HI group. (**D**) Quantification of ipsilateral MBP area loss. *p < 0.05 versus the sham group, ^#^*p* < 0.05 versus the HI group. Data are expressed as the mean value ± SEM, *n* = 6 rats per group.

As mentioned above, aFGF provided short-term (1 day) protection against neonatal HI brain injury. Thus, we investigated the long-lasting (7 day) effects of aFGF treatment. The hemisphere ipsilateral to the ligated side from the HI group exhibited extensive atrophy and brain tissue damage (Figure 6A). However, this damaged brain tissue was significantly decreased in the aFGF group compared to the HI group.

To further quantify the tissue loss caused by HI injury, immunohistochemical staining was performed. We analysed MAP-2 loss as a measure of grey matter damage and MBP loss as a measure of white matter damage at 1 week post-insult [24]. Notably, we observed a significant reduction in MAP-2-positive (52 ± 4%) and MBP-positive area loss (59 ± 6%) in the aFGF-treated group compared to the HI group (Figure 6B, 6C and 6D).

### aFGF inhibits apoptosis and ER stress in oxygen-glucose deprivation (OGD)-treated PC12 cells

**Figure 7:**
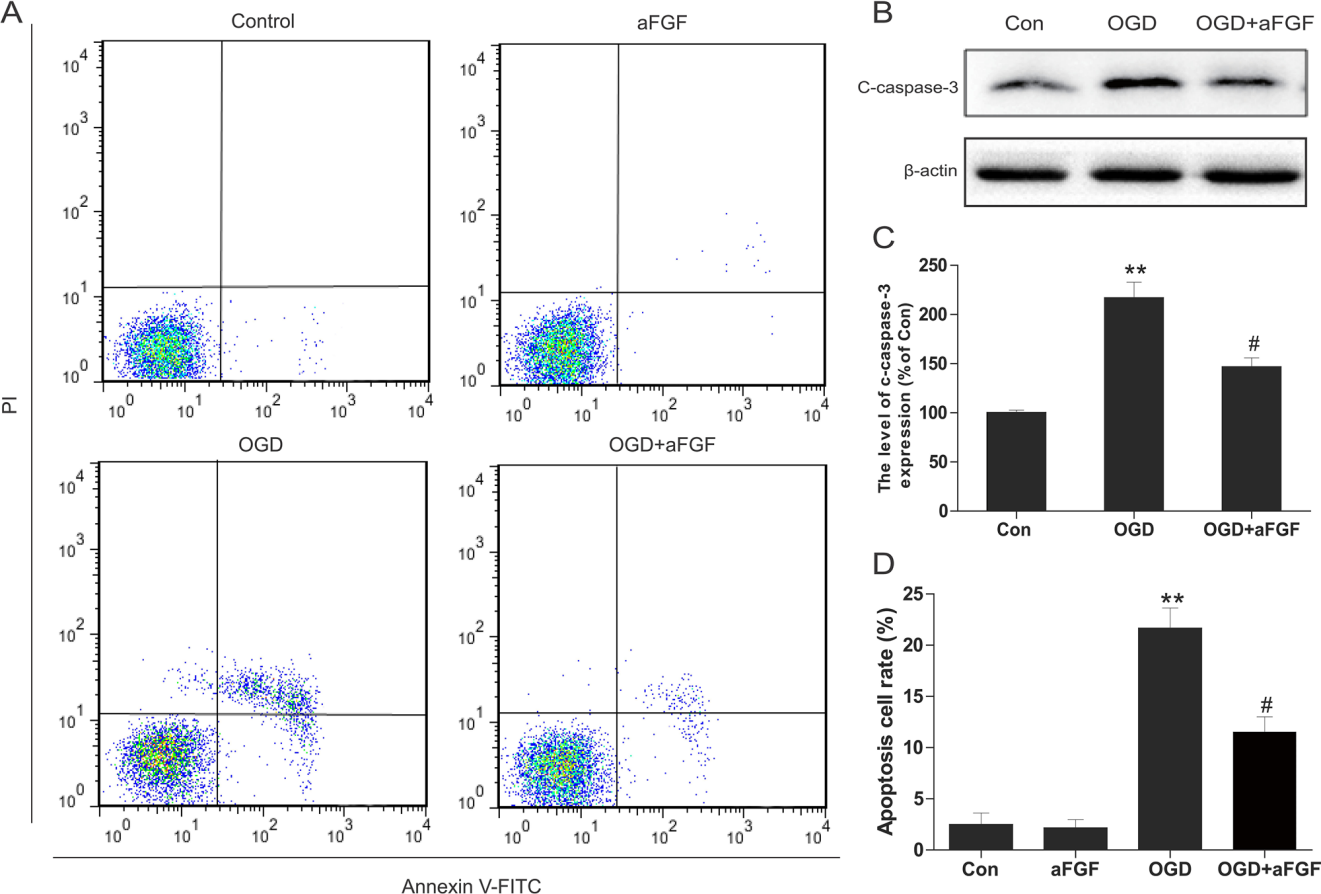
aFGF inhibits OGD-induced apoptosis in PC12 cells (**A**) PC12 cells were pretreated with aFGF for 2 h and then OGD for 24 h. The cells were then stained with annexin V-FITC/propidium iodide and detected via flow cytometry. The right panel indicated the apoptotic cells. (**B**) Representative western blots of cleaved caspase 3 in the Control, OGD and OGD + aFGF-treatment groups. (**C**) Quantification of western blot data from B. **p < 0.01 versus the sham group, ^#^*p* < 0.05 versus the OGD group. (**D**) Bar diagram of apoptotic cell rates from A. **p < 0.01 versus the sham group, ^#^*p* < 0.05 versus the OGD group. Data are expressed as the mean value ± SEM, *n* = 5 per group.

PC12 cells were treated with OGD to mimic the HI model *in vitro*. To test the effects of aFGF on OGD-induced apoptosis in PC12 cells, flow cytometry was performed. After 24 h of OGD, the percentage of apoptosis was significantly up-regulated. However, the apoptosis rate signiﬁcantly decreased in the aFGF group, and aFGF alone did not exert any significant effects (Figure 7A, 7D). We further assessed expression level of apoptosis related protein cleaved caspase 3. Western blot for cleaved caspase 3 revealed a consistent effect of aFGF intervention with our data above. aFGF intervention obviously inhibited the OGD-induced up-regulation of apoptosis in PC12 cells (Figure 7B, 7C).

**Figure 8:**
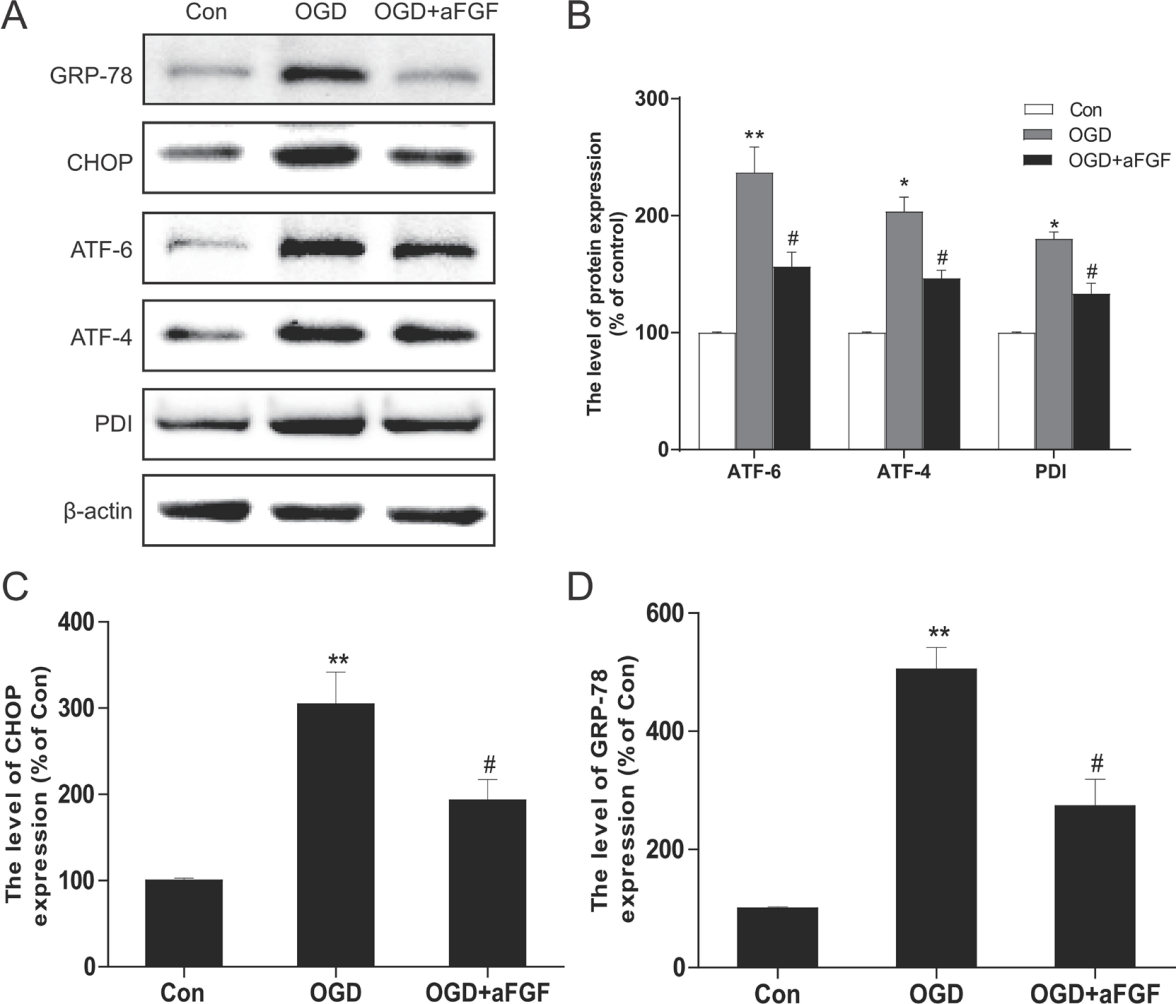
aFGF inhibits OGD-induced ER stress-related protein expression in PC12 cells (**A**) Representative western blots of the ER stress markers GRP-78, CHOP, ATF-6, ATF-4 and PDI in the Control, OGD and OGD + aFGF-treated groups. (**B**), (**C**) and (**D**) Quantification of western blot data from A. *p < 0.05, **p < 0.01 versus the sham group, ^#^*p* < 0.05 versus the OGD group. Data are expressed as the mean values ± SEM, *n* = 5 per group.

In addition, PC12 cells were treated with OGD alone or in combination with aFGF to further test our hypothesis that the effects of aFGF are connected with the inhibition of ER stress in the cellular model. Our data showed that the protein levels of GRP-78, CHOP, ATF-6, ATF-4 and PDI were significantly increased after 24 h of OGD injury and that aFGF markedly prevented these increases at a concentration of 50 ng/ml (Figure 8). Collectively, these data indicated that the protective effects of aFGF might at least partially involve the inhibition of apoptosis and ER stress.

## DISCUSSION

Neonatal HI brain injury is a serious birth complication associated with permanent neurodevelopmental handicaps in the form of cerebral palsy, epilepsy, mental retardation, or learning disabilities, as well as a high societal economic burden [6]. Although the utility of hypothermia with respect to reductions in death and disability is now well established in newborns with HI brain injury [25–27], as many as 40–50% of children with this condition still suffer long-term neurological injury or die [28, 29]. Thus, finding new and more effective treatments are urgently required. The complex pathophysiology of HI brain injury provides multiple targets associated with different time points of the disease process. For instance, in the early stages of the disease, therapies are mainly focused on reducing the oxidative, excitotoxic and apoptotic mediators [30, 31], whereas in the later phase of the disease, stimulation of the neurotrophic properties and reductions in the levels of inflammatory cytokines can promote neuronal and oligodendrocyte regeneration [22, 32, 33].

Our study presents evidence that 1) aFGF treatment effectively inhibits ER stress signalling pathways both *in vivo* and *in vitro*, 2) aFGF treatment significantly reduces neuronal cell apoptosis, and 3) aFGF treatment exerts long-term neuroanatomical effects against HI brain injury. Our results are in keeping with recent findings indicating that aFGF reduces ER stress in different animal models. For example, aFGF inhibited ER stress in Parkinson’s model rats, which were associated with activation of ERK1/2 and PI3K/Akt signalling pathways [34]. Similar effects of aFGF were also observed in diabetes-induced testicular cell model [35]. It was therefore conceivable that aFGF plays an important role in ER stress induced by neonatal HI brain injury. To our knowledge, this is the first report showing inhibition of endoplasmic reticulum stress is involved in the neuroprotective effect of aFGF in neonatal hypoxic-ischaemic brain injury and suggest a promising therapeutic strategy to this disease. The present study lays the ground work for future translational confidence of aFGF in HI brain injury.

Several explanations have been proposed regarding the mechanisms underlying HI-induced ER stress. HI resulted in oxygen and glucose deprivation in neuronal cells, as well as subsequent ATP depletion [36, 37]. The ER functions as an intracellular Ca^2+^ store and plays an important role in Ca^2+^ homeostasis by releasing Ca^2+^ from the ER and pumping Ca^2+^ into the ER lumen via Ca^2+^-ATPase and various receptors. When ATP levels decrease, ER function was suppressed and Ca^2+^ may be depleted, which impairs protein folding in the ER and leads to ER stress [38]. It is universally accepted that the adaptive responses to misfolded proteins in the ER protect cell from death. However, when protein misfolding is excessive or persistent, ER stress triggers cell death, typically apoptosis, leading to secondary injury after HI injury [39]. Apoptosis is an important mechanism of cell death after HI injury in the immature brain, and it has been suggested that in newborns, apoptosis may be several times more pronounced than necrosis [27, 40]. Moreover, there is growing evidence that apoptosis mainly occurs in the early stage of HI brain injury and can last for days or even weeks [41]. As Badiola reported, at least 50% of dying cells showed morphologic feature of apoptosis in the OGD–treated cortical neurons [42]. Several mechanisms linking ER stress to cell death have been proposed, including direct activation of kinases, proteases, Bcl-2 family proteins and transcription factors, et al [43]. To determine the possible mechanisms by which aFGF attenuates neonatal HI brain injury, we studied ER stress pathway proteins via immunofluorescence and western blotting. Our results showed that HI resulted in strong UPR activation and that aFGF significantly reduced the levels of UPR-related proteins following a single administration of 100 ng/g aFGF. Furthermore, aFGF administration attenuated neuronal cell death and exerted long-term protective effects. In the *in vitro* model, we mimicked HI by introducing OGD in PC12 cells and observed that aFGF prevented cell death partially by inhibiting ER stress.

Treating central nervous system (CNS) diseases was very difficult due to the ability of the blood–brain barrier (BBB) to severely limit the entry of all but the non-polar and smallest compounds [44]. Intracerebroventricular, intraparenchymal and intrathecal injections are capable of delivering therapeutic agents directly to the CNS, but these routes of administration are invasive and are likely not practical for drugs that need to be given chronically [45]. The characteristic of intranasal administration that enables biologics, such as proteins, peptides, oligonucleotides, viral vectors, and even stem cells bypassing the BBB to the CNS has lead intranasal route of administration to be applied in many recent trials. Therefore, in our study, we chose the non-invasive intranasal administration method. Moreover, to increase drug delivery efficiency, hyaluronidase has been used as a permeation enhancer before aFGF administration due to its capacity to increase vascular permeability, reduce biological fluid viscosity and render tissues more accessible to certain drugs [46, 47].

There are certainly limitations to use aFGF as a therapy for HI brain injury, and these limitations still require further investigation and improvement. It is disappointing that despite the numerous drugs that have proven to be beneficial in animal models, hypothermia is now the only established standard treatment for neonatal HI brain injury in humans. This gap may be due partly to the limitations of preclinical studies. In addition, few species and strains of animals whose brains and responses to brain injury closely resemble those newborn humans are available. Since neonatal HI brain injury is a complicated injury, target only one pathophysiological pathway is far from sufficient to combat this disease. Promising therapeutic drugs should be studied together with hypothermia because the pharmacokinetics and pharmacodynamics of various drugs can be greatly altered when temperature changes. Moreover, the 7-day outcomes used in this study may not be long enough to determine the effectiveness of HI injury therapy, long-term studies lasting 2–3 weeks or even longer may be necessary to draw more substantive conclusions. Besides, future *in vitro* assays regarding the effects of aFGF on primary neurons may be helpful in elucidating the underlying mechanisms of these effects. Nevertheless, the neuroprotective effects of aFGF on HI brain injury are confirmed. Our study demonstrated that aFGF therapy may be suitable for treating central nervous system diseases caused by HI injury.

## MATERIALS AND METHODS

### Reagents and antibodies

All reagents used in this study were commercially available. aFGF was purchased from Key Laboratory of Biotechnology and Pharmaceutical Engineering, Zhejiang, China, and the *In Situ* Cell Death Detection Kit was purchased from Roche (South San Francisco, CA, USA). Foetal bovine serum and Dulbecco’s modified Eagle’s medium (DMEM) were purchased from Invitrogen (Carlsbad, CA, USA). Antibodies against ATF-6, ATF-4, GRP-78, PDI, XBP-1 and cleaved caspase 12 were purchased from Abcam (Cambridge, MA, USA). Anti-β-actin, CHOP, microtubule-associated protein 2 (MAP-2) and myelin basic protein (MBP) antibodies were purchased from Santa Cruz Biotechnology (Santa Cruz, CA, USA). Anti-cleaved caspase 3 antibody was purchased from Cell Signaling Technology (Danvers, MA, USA). The appropriate secondary antibodies were purchased from Abcam or Santa Cruz Biotechnology.

### Animals and surgical procedures

All animal procedures were performed in accordance with the guidelines for the Care and Use of Laboratory Animals from the National Institutes of Health Experimental Animal Laboratory and approved by the Laboratory Animal Ethics Committee of Wenzhou Medical University. Efforts were made to minimize animal suffering and to minimize the number of animals used. The HI model was based on the modified Rice-Vannucci method [48] and produced on postnatal day 7 (P7) male and female Sprague–Dawley (SD) rat litters whose brain is histological similar to that of a 32–34 week gestation human fetus or newborn infant [49]. Briefly, pups were anesthetized with diethyl ether, and the left common carotid artery was ligated. After a 2 h recovery period, the pups were placed in a glass chamber containing a humidified atmosphere of 8% oxygen/92% nitrogen which was submerged in a 37°C water bath to maintain normothermia. Pups were kept in the hypoxic chamber for 2.5 h and then returned to their dam. For control measurements, we included a sham group that had a ligature placed in a fashion identical to that described above, although this procedure did not entail actually occluding the vessel or causing hypoxia. The death rate of our model was zero.

### aFGF administration

The intranasal route allows small molecules to rapidly enter the cerebrospinal fluid from the nasal cavity and subsequently distribute to the brain and spinal cord [50]. aFGF was freshly prepared by dissolving and diluting in sterile 0.9% saline solution at a concentration of 100 μg/ml and stored at −20°C. Rats were randomized to a vehicle (saline) or an aFGF group. Saline or aFGF was administered immediately after hypoxia and then twice a day until postnatal day 14 (P14). Before administering aFGF intranasally, each nostril was treated with 3 μl of hyaluronidase (100 U, Sigma-Aldrich, St. Louis, MO) in PBS to increase the permeability of the nasal mucosa. Thirty minutes later, pups received 100 ng/g of aFGF (HI+aFGF group) twice in each nostril, while the HI group received saline only. The rats were held ventral-side up, and a small-modified 27-French catheter was inserted into either nare. Saline or aFGF was slowly administered, and the rats were held for 1–2 min to ensure absorption. The doses were spaced 5–10 min apart, and a total dose of 100 ng/g was administered [50].

### Measurement of infarct ratio

At 24 h after HI, the pups were decapitated, and their infarct ratios were measured in a blinded fashion. Briefly, their brains were quickly removed and maintained at −80°C for 5 min and then cut at 2 mm intervals into 5 coronal sections, beginning at the frontal pole. After incubation in 1% 2, 3, 5-Triphenyltetrazolium chloride (TTC) solution (Sigma-Aldrich, St. Louis, MO) for 15–20 min at 37°C, the brain slices were kept in 4% paraformaldehyde for 30 min. Infarct volumes were analyzed using the Image J software (http://imagej.nih.gov/ij/) by outlining both hemispheres on full section images. The index of infarct was calculated as the ratio of the overall infarct volume to the total brain volume.

### HE and Nissl staining

Tissue sections were initially subjected to HE staining and Nissl staining for general assessment of histopathology changes. Freshly cut, free-floating brain sections (5 μm thick) were prepared from P14 rats. Then, the slices were mounted onto slides and stained using a Haematoxylin and Eosin Staining Kit (Beyotime) or Nissl staining solution (Beyotime) for 10 min. Quantitation was performed by counting the number of normal neurons in five randomly chosen fields within each slide at 400× magnification using a Nikon ECLIPSE Ti microscope (Nikon, Tokyo, Japan). The counting was conducted in a blinded fashion and the average cell density was calculated as the ratio of the overall normal neurons number to the total area of the chosen field.

### TUNEL staining

To measure apoptosis-like cell death, terminal-deoxynucleotidyl transferase mediated nick end labeling (TUNEL) staining was performed using *In Situ* Cell Death Detection Kit at 7 d after HI injury. According to manufacturer’s protocols, brain sections (5 μm thick) were deparaffinized, rehydrated and incubated in a 20 μg/ml proteinase K working solution for 15 min at 37°C. Then, sections were incubated with TUNEL reaction mixture in a dark humidified chamber for 1 h at 37°C, followed by a final wash for 3 × 5 min with phosphate buffered solution (PBS). Finally, the sections were treated with 4, 6-diamidino-2-pheny-lindole (DAPI) for 5 min at room temperature and mounted with Mowiol [51]. Quantitation was performed by counting the number of positive cells in five randomly chosen fields within each slide at 400× magnification using a Nikon ECLIPSE Ti microscope. The counting was conducted in a blinded fashion and the percentage of TUNEL positive cells was expressed as the number of TUNEL-stained nuclei divided by the total number of DAPI-stained nuclei.

### Immunofluorescence staining

Brain tissue samples obtained from the animals 24 h after HI injury were kept in 4% paraformaldehyde for a post-fix overnight. After dehydration, the brains were embedded in paraffin, and sliced coronal in 5-μm. Sections were deparaffinized, rehydrated and incubated with 5% bovine serum albumin for 30 min in a 37°C oven, followed by incubation with primary antibody at 4°C overnight. The primary antibody dilutions were 1:500 for anti-GRP78 (ab21685, Abcam) and 1:100 for anti-CHOP (sc-793, Santa Cruz Biotechnology). The sections were incubated with Alexa-Fluor 488-conjugated donkey anti-rabbit antibody (1:1000; Invitrogen Corporation, Carlsbad, CA, USA) for 1 h at 37°C and then washed three times with 1×PBS. Finally, tissues were counterstained with DAPI for 5 min and mounted with Anti-fade Mounting Medium. Negative controls were performed using the same procedure described above in the absence of the primary antibody [52]. All images were captured at 600× magnification using a Nikon ECLIPSE Ti microscope.

### Immunohistochemistry and antibodies

Coronal paraffin sections (5 μm) of paraformaldehyde-fixed brains were dewaxed in xylene overnight and rehydrated in ethanol and distilled water. Endogenous peroxidase activity was then blocked with 3% H_2_O_2_ (30% H_2_O_2_ diluted 80% in methanol) for 30 min. Antibodies (MAP-2, sc-20172, 1:200, Santa Cruz Biotechnology; MBP, sc-13914, 1:200, Santa Cruz Biotechnology) were diluted in 10 mM PBS containing 1% bovine serum albumin. Then, the sections were incubated with primary antibodies at 4°C overnight followed by biotinylated donkey anti-rabbit (1:1000; Abcam) or donkey anti-goat (1:300; Santa Cruz Biotechnology) secondary antibodies at 37°C for 1 h. Binding was visualized with 3,3′-diaminobenzidine (DAB) staining. The images of MAP-2 and MBP were captured with a Nikon ECLIPSE Ti microscope at 40× magnification. Area loss was measured as 1-(ipsi-/contralateral MAP-2 or MBP-positive area) and quantified using Image J software (http://imagej.nih.gov/ij/).

### Cell culture and treatments

PC12 cells were obtained from the American Type Culture Collection of the Chinese Academy of Sciences, Shanghai, China. Cells grown in DMEM (4.5g/L glucose) were supplemented with 10% foetal bovine serum and 1% antibiotics and were maintained at 37°C in a humidified incubator with 5% CO_2_/95% room air. The cells subcultured into 60-mm or 35-mm dishes were exposed to a hypoxia chamber (1029; Thermo Fisher Scientific, Waltham, MA, USA) with oxygen concentration < 0.2% for 24 h. aFGF was freshly prepared by dissolving and diluting in PBS. Two hours before OGD, the culture medium was changed to a low-sugar DMEM (1g/L glucose) and cells were added with aFGF (50 ng/ml). For the control group, PBS alone was used.

### Flow cytometry analysis

To quantify the apoptotic rate of the PC12 cells treated with OGD or aFGF, cells and medium were harvested after treatment. The cells were re-suspended in 1× binding buffer and stained with a PI/Annexin V-FITC kit (Invitrogen, Carlsbad, CA, USA) in the dark for 15 min, and then analysed by a FACScan flow cytometer (Becton Dickinson, Franklin Lakes, NJ, USA), according to the manufacturer’s instructions.

### Western blot analysis

For *in vivo* protein analysis, the cerebral hippocampus of each rat was dissected 24 h after HI injury and immediately stored at −80°C for western blotting. Brain tissues were homogenized in modified buffer [50 mM Tris-HCl, 1% NP-40, 20 mM dithiothreitol, 150 mM NaCl (pH 7.4)] containing protease inhibitor cocktail (10 μl/ml; GE Healthcare Biosciences, Piscataway, NJ, USA). As for *in vitro* protein analysis, PC12 cells were lysed in RIPA buffer (25 mM Tris-HCl, 150 mM NaCl, 1% Nonidet P-40, 1% sodium deoxycholate, and 0.1% sodium dodecyl sulfate) with supplemental protease and phosphatase inhibitors (GE Healthcare Biosciences, Piscataway, NJ, USA). The complex above was then centrifuged at 12,000 rpm, and the supernatant was obtained for protein assay. After centrifugation, the extracts above were quantified with bicinchoninic acid (BCA) reagents (Thermo, Rockford, IL, USA). Proteins (60 μg) were subjected to sodium dodecyl sulphate polyacrylamide gel electrophoresis (SDS-PAGE; Bio-Rad, Hercules, CA) using 12% gels and blotted on to nitrocellulose membranes. After blocked with 5% milk (Bio-Rad) in TBS with 0.05% Tween 20 (TBST) for 1.5 h, these membranes were then incubated with the following antibodies at 4°C overnight: GRP-78 (ab21685, 1:1000, Abcam), ATF-6 (ab122897, 1:1000, Abcam), PDI (ab2792, 1:1000, Abcam), ATF-4 (ab50546, 1:1000, Abcam), XBP-1 (ab37152, 1:1000, Abcam), CHOP (sc-793, 1:300, Santa Cruz Biotechnology), cleaved caspase 3 (9664S, 1:1000, Cell Signaling Technology) and cleaved caspase 12 (ab21685, 1:1000, Abcam). Afterwards, horseradish peroxidase (HRP)-conjugated secondary antibodies (1:3000, Santa Cruz Biotechnology) were incubated with the blots for 1 h at room temperature. Western blotting of β-actin (sc-7160, 1:300, Santa Cruz Biotechnology) was performed to demonstrate equal protein loading, and used as an internal control [53]. Signals were visualized via a ChemiDocXRS + Imaging System (Bio-Rad). Each sample was analyzed in 3 times in independent experiments, and the densitometric values of the bands obtained via Quantity One software were subjected to statistical analysis.

### Statistical analysis

All data were expressed as the mean ± SEM of three independent experiments. Student’s *t* test or factorial analysis of variance with Tukey post hoc comparisons was performed. *P* < 0.05 was considered statistically significant. All statistical analyses were performed using GraphPad Prism 5 for Windows.
